# Supplementary material for: Health care workers’ perceptions and bias toward men as HIV clients in Malawi and Mozambique: A qualitative study
Source: PLOS Glob Public Health. 2023 Oct 24;3(10):e0001356. doi: 10.1371/journal.pgph.0001356 (PMC10597488; doi:10.1371/journal.pgph.0001356)
Supplement: S1 Text — (DOCX) [file pgph.0001356.s001.docx]

**S1 Text**

**Focus Group Guide – Uptake - English version**

*Note: Focus Groups will be open-ended and guided by group answers. Focus Groups are meant to help us understand barriers and facilitators to ART uptake as understood by healthcare providers. We are particularly interested in multi-level barriers to care – including individual-, community-, and facility-level.* *The following questions are meant to guide interviewers. This outline reflects a general guide but actual Focus Group discussions will vary.*

I would like to talk to you about your experience with HIV services based on your experience at this facility. With the Test and Treat policy there may be more people who can start ART but feel healthy. I would like to understand what helps these people start ART.

1. Do you think people who feel healthy have a harder time starting ART than people who feel sick? Why/why not?
   1. Is there anything within a person that makes it difficult for healthy clients to take up ART (individual-level)?
   2. Is there anything within the community that makes it difficult for healthy clients to take up ART (community-level)?
   3. Is there anything at the health facility that makes it difficult for healthy clients to take up ART (facility-level)?
2. From your experience, do you have any concerns about starting ART for people who feel healthy?

*Prompt:*

1. *Fear of disclosure*
2. *Lack of information*
3. *No time to travel to the facility*
4. *ART side effects*

*NOTE: We want to get more information than “Accepting status”*

1. From your experience, what helps people who feel healthy to start ART?

Is this different for people who are already sick?

- 1. Is there anything within a person that helps healthy clients to take up ART (individual-level)?
  2. Is there anything within the community that helps healthy clients to take up ART (community-level)?
  3. Is there anything at the health facility that helps healthy clients to take up ART (facility-level)?

1. What do you think the facility could do to facilitate ART uptake for healthy populations who are not pregnant?
